# The Screening and Evaluation of *Fucus serratus* and *Fucus vesiculosus* Extracts against Current Strains of MRSA Isolated from a Clinical Hospital Setting

**DOI:** 10.1038/s41598-019-54326-4

**Published:** 2019-11-29

**Authors:** Annabel Higgins Hoare, Shiau Pin Tan, Peter McLoughlin, Patrick Mulhare, Helen Hughes

**Affiliations:** 1 Waterford Institute of Technology, Cork Road, Waterford, Ireland; 2 University Hospital Waterford, Ardkeen, Waterford, Ireland

**Keywords:** Screening, Chemical biology

## Abstract

Antimicrobial resistant strains of infection are afflicting clinical settings, driving the search for novel antimicrobial compounds. Naturally sourced bioactives, for instance those from seaweeds, have the potential to ameliorate this issue. As such, solvent extracts from the edible Irish seaweeds *Fucus serratus* and *Fucus vesiculosus* were screened for antimicrobial activity against 28 clinically isolated strains of MRSA, including one GISA (glycopeptide intermediate *S. aureus*) and two mecC gene containing strains. The water extract of *F. vesiculosus* was the most promising extract went on to be tested for biofilm prevention and disruption activity. The disk diffusion method was used to investigate the inhibition of the bacterial pathogens tested while MIC, MBC and biofilm disruption and prevention analyses were performed spectroscopically and by plate counts, respectively. Solvent extracts were found to have a wide array of antimicrobial activity against the strains tested, with the water extract from *Fucus vesiculosus* being the most promising. This extract was also found to both prevent and disrupt MRSA biofilms indicating the potential extract as new antimicrobials, and raising the possibility of their possible use in therapeutics.

## Introduction

Seaweeds, benthic marine macro algae which can typically be found at various levels of beach and sea depth, have come under scrutiny for the bioactive compounds which they are known to possess. They have been used for millennia by people to aid numerous dietary and medicinal needs^1^. Due to their autotrophic nature^2^, seaweeds have evolved to produce an assortment of bioactive compounds; from the simple resources found in the marine environment to compounds used for the purpose of self-preservation in harsh competitive environments^3^. These bioactives can potentially be extracted from seaweeds and utilised for a range of applications; from supplements to pharmaceuticals, with the industrial potential for bioactives of natural origin being vast^4–11^.

As bacteria have evolved to reject various established antibiotics, infections are becoming more serious leading to increased treatment cost and a higher risk of complications associated with the treatment of once trivial infections. Antimicrobial resistant (AMR) infections have inspired a plethora of research into finding novel antibiotics to which AMR strains of infections are susceptible. A 2016 review commissioned by the British Prime Minister David Cameron projected that by 2050 AMR will result in 10 million deaths per annum^12^. Findings such as this have encouraged scientists to look for potentially novel antimicrobial activity from natural products.

Many of the bioactive natural products generated by organisms involve complex and poorly understood mechanisms of action. These compounds may be novel and as such have a greater chance of a novel activity against the more dangerous strains of infection. Antimicrobials from natural products could have the ability to revolutionise the treatment of modern multi-resistant infections.

Methicillin-resistant *Staphylococcus aureus* (MRSA) is a multidrug resistant strain of the common *Staphylococcus aureus*. *S. aureus* exists parasitically on the skin of most healthy individuals. However, at some point in its life cycle, infection can occur in immuno-compromised people. Resistant strains of *S. aureus* were noted after just a decade subsequent to the introduction of penicillin in the 1940’s with the first methicillin-resistant strain emerging at the end of the 1960’s^13^. The label of MRSA as a β-lactum antibiotic resistant strain has stuck since its emergence, regardless of the fact that oxacillin and/or cefoxitin are now used as susceptibility markers for penicillin based antibiotics^14^. More recently, MRSA strains with resistance to a variety of antibiotics with a range of modes of action (such as the clinical strains tested as part of this paper) have become widespread.

Further to the problems arising from AMR, MRSA is also a producer of biofilms which are a polymeric matrix excreted by bacteria that aggregate for their inhabitation and their improved adherence to surfaces. Bacteria produce biofilms for a myriad of reasons, however, this optimisation of their environment can make bacterial infections difficult to treat^15^. Their ability to make infections more resilient against mechanical debridement^16^ and tolerant to antimicrobials^17^ make biofilm producing pathogens more challenging to treat.

This study tested extracts from the brown seaweeds *Fucus serratus* and *Fucus vesiculosus* found on the coast of Wexford, Ireland against 28 clinically isolated MRSA samples found in a hospital environment during 2016. The most promising of these samples underwent minimum inhibitory concentration (MIC) and minimum bactericidal concentration (MBC) assays in addition to assays for biofilm prevention and disruption activity.

## Results

### Solvent extraction yields from the seaweeds F. serratus and F. vesiculosus

The yield of extracts generated from the seaweeds *F. serratus* and *F. vesiculosus* harvested in September 2015 from different solvents at a ratio of 1:100 are displayed in Table 1.

**Table 1:** Percentage of crude extract yields for solvents of decreasing polarity (n = 3).

% of Extract				
	Water	Methanol	Acetone	Ethyl. Acetate
Polarity Index	10.2	5.1	5.1	4.4
*Fucus serratus*	32.51 ± 1.57^a^	13.15 ± 1.70^b^	5.59 ± 0.74^c^	3.96 ± 0.28^d^
*Fucus vesiculosus*	28.31 ± 1.54^e^	12.93 ± 1.21^b^	5.81 ± 0.55^c^	3.96 ± 0.39^d^

Data (n = 3) are presented as the mean ± SD; Data that do not share a common superscript are statistically different (*ρ* < 0.05; One-way ANOVA followed by *post-hoc* analysis using Tukey’s multiple comparison test).

These extractions found that the quantity of crude extract from the seaweed increased with an increase in solvent polarity. This is not an unexpected result as carbohydrates, which are polar, account for approximately 20–50% of dried seaweeds^18^. Proteins (3–11% dry mass^19^) and lipids (1–6% dry mass^20^) can also have varying degrees of polarity, as such, some can be solubilised by polar solvents while the remainder will be either solubilised by non-polar solvents or be completely insoluble.

### Antimicrobial activity of crude seaweed extracts

The eight crude seaweed extracts were tested for antimicrobial activity against a range of MRSA strains donated by University Hospital Waterford in order to establish the extract with the best inhibition against clinical MRSA strains. The results of the anti-MRSA screen are displayed in Tables 2 and 3.

**Table 2:** Antimicrobial activity of 5 mg crude extracts of *F. vesiculosus* against various MRSA strains using the disk diffusion method.(n = 3).

MRSA Strain	Ethyl Acetate	Acetone	Methanol	Water
618	+	++	++	+
619	+	++	++	++
620	++	++	+	+
621	++	++	+	+
666	+++	+++	+++	++++
667	+++	+++	+	++++
668	+++	++++	++++	+++++
669	+++	++++	++++	+++++
670	++	+++	+++	++++
671	++	+++	++++	++++
672	+++	+++	++	+++
673	+++	+++	+++	+++++
674	++	+++	+++	++++
675	++	++	++	+++
676	++++	+++++	++++	+++++
677	++	++	+	++
678	++	++	++	++++
679	+++	+++	+++	+++++
680	+++	++	++	++
681	++	+++	+++	+++++
682	++++	+++++	++++	+++++
683	+++	+++	+++++	+++++
684	−	+	+	++
685	−	++	++	++
686	+++	+++	+++	++++
687	−	+	−	++
688	−	−	+	++
689	−	++	++	+++
Positive control^a^	++++	++++	++++	++++
Negative control^b^	−	−	−	−

^a^Chloramphenicol antibiotic disk - 10 µg/disk. ^b^Negative control - 50 µL of specific solvent. Inhibition zone reported as diameter of clear inhibition (including 6 mm disk) in mm; - indicates no inhibition, + indicates inhibition zone of 6 mm–9.9 mm, ++ indicates inhibition zone of 10 mm–14.9 mm, +++ indicates inhibition zone of 15 mm–19.9 mm, ++++ indicates inhibition zone of 20 mm–24.9 mm, +++++ indicates inhibition zone of >25 mm.

**Table 3:** Antimicrobial activity of 5 mg crude extracts of *F. serratus* against various MRSA strains using the disk diffusion method. (n = 3).

MRSA Strain	Ethyl Acetate	Acetone	Methanol	Water
618	−	+	−	−
619	−	+	−	++
620	−	+	+	−
621	−	−	+	−
666	−	−	+	+
667	−	−	−	+
668	+	+++	+++	+++
669	−	++	+++	++
670	−	−	−	+
671	−	+	−	+
672	−	+	−	+
673	−	++	++	++
674	−	+	++	+
675	−	−	++	+
6v76	−	+++	++	+++
677	−	−	−	+
678	−	−	+	+
679	−	++	++	+++
680	−	−	−	+
681	−	−	++	++
682	−	++	++	+++
683	−	++	++	+++
684	−	−	+	+
685	−	−	++	++
686	−	+	+	+
687	−	−	−	+
6−88	−	−	−	+
689	−	+	−	++
Positive control^**a**^	++++	++++	++++	++++
Negative control^b^	−	−	−	−

^a^Chloramphenicol antibiotic disk - 10 µg/disk. ^b^Negative control - 50 µL of specific solvent. Inhibition zone reported as diameter of clear inhibition (including 6 mm disk) in mm; - indicates no inhibition, + indicates inhibition zone of 6 mm–9.9 mm, ++ indicates inhibition zone of 10 mm–14.9 mm, +++ indicates inhibition zone of 15 mm–19.9 mm, ++++ indicates inhibition zone of 20 mm −24.9 mm, +++++ indicates inhibition zone of >25 mm.

The crude solvent extracts for *Fucus vesiculosus* can be seen to contain more anti-MRSA activity than those of *Fucus serratus*. Activity was also found to increase with an increase in solvent polarity for most of the extracts, indicating that the compound(s) responsible for anti-MRSA activity are on the polar side of the spectrum.

*Fucus vesiculosus* extracts displayed a wide array of activity against the clinical MRSA strains used in this experiment. The ethyl acetate extracts displayed the least amount of activity, with no inhibition at all for two of the 25 mecA strains, the GISA and neither of the two mecC strains. Acetone, methanol and water extracts all inhibited every one of the mecA strains with water showing the best inhibition. This, in addition to the water extracts being active against both the GISA and the two mecC strains of MRSA, resulted in the water extract from *Fucus vesiculosus* being deemed the most promising.

While crude water extracts for *Fucus serratus* demonstrated the most potent activity, the methanol and acetone extracts also exhibited antimicrobial activity against the various strains of MRSA. Between the acetone, methanol and water extracts for *Fucus serratus*, there was some degree of inhibition for all the strains of MRSA. Water extracts of *Fucus serratus* presented with antimicrobial activity against both the GISA and the mecC strains of MRSA (687–689 strains), indicating potential for this extract to be used as an antimicrobial against these particularly troublesome strains.

### Minimum inhibitory concentration (MIC), minimum bactericidal concentration(MBC), biofilm prevention and biofilm disruption analysis

**Figure 1:**
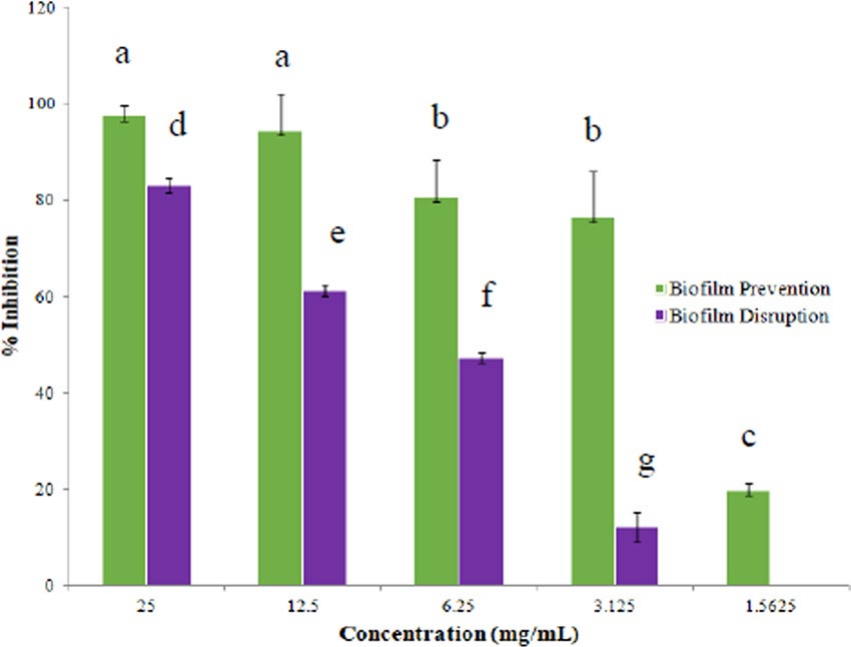
Biofilm prevention and disruption (%) of MRSA (676) by water extract of *Fucus vesiculosus* over a series of twofold dilutions in concentration (mg/mL) using the crystal violet assay (n = 9). Data points which do not share a letter are significantly different (ρ ≤ 0.05, using Tukey one-way analysis).

**Figure 2:**
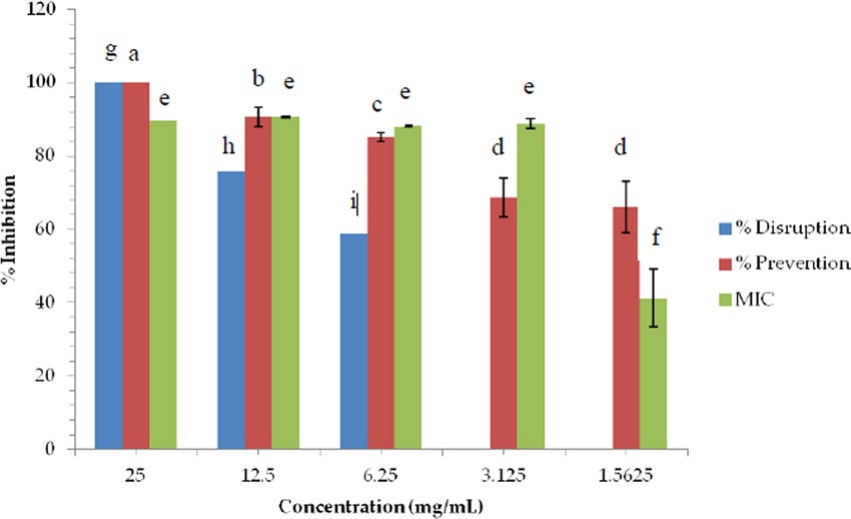
Inhibition (%) of MRSA (676) by water extract of *Fucus vesiculosus* over a series of twofold dilutions in concentration (mg/mL) (n = 18). Data points which do not share a letter are significantly different (ρ ≤ 0.05, using Tukey one-way analysis).

The concentrations of the water extract from *Fucus vesiculosus* required for MIC, MBC, biofilm prevention and biofilm disruption by both cell viability and the crystal violet assay are outlined in Table 4 and further in Figs. 1 & 2. These concentrations were found to inhibit/disrupt at least 80% of MRSA (676) which was used as an indicator in this experiment.

**Table 4:** Concentrations (mg/mL) of water extract from *Fucus vesiculosus* required to inhibit 100% growth for MBC and >80% of MRSA (676) for biofilm disruption, prevention and MIC (n = 18) (±standard error).

Concentration (mg/mL)	Disruption	Prevention	MIC	MBC
Cell viability assay	25 ± 1.06	6.25 ± 0.31	3.125 ± 0.21	25
Crystal violet assay	12.25 ± 0.47	3.125 ± 3.14	—	—

The crystal violet assay for biofilm disruption and prevention determined that a concentration of 12.25 and 3.125 mg/mL respectively was required to inhibit >80% of the biofilm.

The cell viability assay however, established that a dose of 25 mg/mL of water extract from *Fucus vesiculosus* will result in the disruption of a biofilm formed by an overnight culture of MRSA (676) adjusted to an optical density (625 nm) equivalent to 0.5 McFarland Standard or 10^7^–10^8^ cfu/mL. Similarly, 25 mg/mL will have a bactericidal effect on the adjusted culture indicating that this dose is sufficient to completely kill the bacteria. The MIC was found to be 3.125 mg/mL. This concentration of extract will inhibit at least 80% of the culture.

It was determined that a lower concentration of 6.25 mg/mL was required to prevent the formation of a biofilm with MRSA (676).

## Discussion

The high level of activity displayed by extracts and compounds from *Fucus vesiculosus* has been documented in previous studies^21–23^. For example a polyhydroxylated fucophloroethol extract from *Fucus vesiculosus* was found to inhibit the growth of *S. aureus*^24^ among other strains, which shows potential for its’ use against MRSA. Fucoidan, which can be extracted from *Fucus vesiculosus*, has been shown to have antimicrobial effects against oral bacteria including *S. aureus*^21^ and has also been proven to have a synergistic effect against MRSA when administered with the antibiotics oxacillin or ampicillin, increasing the efficacy of treatments by more than four times^25^. There is little evidence of the antimicrobial nature of extracts of *Fucus serratus*. One study noted no inhibition of either *Fucus serratus* or *vesiculosus* against *S. aureus* or *E.coli*, however this study was performed on the seaweed itself and not on an extract^26^. There is evidence that bacteria found on the surface of these seaweeds can produce compounds with a microbial antagonistic effect^27,28^, however the method of preparation of these extracts, as well as autoclave studies not detailed here, rule out the possibility of activity from marine-associated bacteria.

From an exhaustive search of literature for activity using extracts from natural sources against GISA strains, it was found that one other study by Haste *et al*. investigated and noted the efficiency of marinopyrrole; a bioactive marine natural product which is not reported as present in the *Fucus* species, against GISA strains^29^. GISA strains refer to *S. aureus* with reduced susceptibility to glycopeptides such as vancomycin, whose mode of action relies on the disruption of peptidoglycan synthesis by binding to the d-alanyl-d-alanine at the free carboxyl end of the peptidoglycan stem peptide^30,31^. GISA strains have been reported more frequently in recent times. A 2001 study by J. Liñares reported just 10 cases of GISA infection worldwide^32^. A more recent 2016 survey of *S. aureus* strains isolated from burn wound patients in Bangladesh reported that 28% of the 40 isolates tested were vancomycin resistant^33^. As such, the susceptibility of the GISA strain of MRSA (689) to the water and acetone extract of both *Fucus serratus* and *Fucus vesiculosus* and the methanol extract of *Fucus vesisucosus*, detailed in Tables 3 and 4, implicates the potential of these extracts for use against AMR infections. The MIC of 3.125 mg/mL for the water extract of *F. vesiculosus* is not as potent as other natural product extracts such as divaricatic acid from *Evernia mesomorpha* which had an MIC of 32 µg/mL^34^. However, it should be noted that the water extract from *F. vesiculosus* is crude, and potency could improve on purification.

Marine ecosystems such as seaweed are a novel source of biofilm disrupting and prevention compounds^35^. The antibiofilm nature of the water extract from *F. vesiculosus* found as part of this study has been noted in several studies. Further antifouling effects of *F. vesiculosus* extracts (of which antibiofilm is included as anti-settlement) have been established^36,37^ indicating that these results are in line with other studies. Interestingly, fucoidan (which can be extracted from *F. vesiculosus*) at a concentration of 250 µg/mL was found to completely suppress the growth of planktonic bacteria and biofilm formation of some orally sourced Gram positive bacteria^21^. Again, this could indicate the potential of seaweed extracts to be used as a complimentary therapy in conjunction with established antibiotics.

Both the crystal violet assay and the cell viability assay were used to determine the biofilm preventative and disruptive action of the water extract from *F. vesiculosus*. Both of these methods were used as, although cell viability is a more accurate measure of antimicrobial activity, this method lacks the specificity to distinguish between biofilm encapsulated bacteria and attached bacteria. As was demonstrated in Table 4, the crystal violet assay determined that a lower dose was required to inhibition >80% of the biofilm, indicating that the cell viability assay is indeed less specific.

The biofilm disrupting and preventing nature of the water extract from *F. vesiculosus* could potentially be an indication of the mode of action of the antimicrobial present in the seaweed, as biofilm producing bacteria are notoriously more difficult to treat using antibiotics^38^. This could pave the way for a potential application of *F. vesiulosus* extracts in tandem with a lower dose of antibiotics. This and the susceptibility of AMR strains of *S. aureus*, coupled with the safe use of seaweed edibles for millennia^1^, make these extracts particularly promising in terms of therapeutics.

Water, being a ‘green’ solvent results in no toxic waste or expensive disposals and is, as such, a desirable solvent to industries such as the pharmaceutical industry. The display of bioactivity exhibited by the water extracts of both *F. vesiculosus* and *F. serratus* in this study shows promise for these extracts to be used as a drug or supplement in a drug delivery system.

Due to the increased risk of infection, active wound dressings containing an antimicrobial may decrease the overall treatment time and cost of burn wound victims. The merit of the tested extracts’ antibiofilm activity in conjunction with its’ antimicrobial effects, could further aide in the treatment of wounds by protecting them against biofilm formation. This could establish an increased vulnerability of the infection to antimicrobial treatment in addition to possibly preventing the uncomfortable debridement of the wound.

## Materials and Methods

### Harvesting and preparation of seaweeds

The two seaweed species *Fucus vesiculosus* and *Fucus serratus* were harvested at Fethard-on-Sea, Co. Wexford, Ireland (52°11′53,68″N, 6°49′34,64″W) in September 2015, these seaweeds were chosen based on antimicrobial activity previously noted for these seaweeds from the same beach^39^. The seaweeds were rinsed and cleaned of any epiphytes and debris. The cleaned seaweeds were then rinsed in distilled deionised water (SG Water Germany) before blot drying excess water from the surface of the seaweeds. Seaweeds were freeze dried (VirTis, SP Scientific, PA, USA) and stored in a sample bag under nitrogen before being processed by blending and sieving until the particle size was less than 850 µm. The seaweed powder was placed in a sample bag under nitrogen and kept at −20 °C for storage until further analysis. This method was established by work previously carried out in WIT^39–41^.

### Production of crude seaweed extracts

Seaweed powder was extracted into various solvents by solvent extraction using a shaking incubator at room temperature (19 °C) for 2 h at a speed of 200 rpm at a ratio of 1:100 (sample:solvent, *w/v*). The solvents used were deionised water, methanol (analytical grade), ethyl acetate (99.5% HPLC grade) and acetone (99.8% HPLC grade). All the solvents used were purchased from Fisher Scientific, Dublin, except deionised water (SG Water, Germany). Extracts were then filtered using Whatman No. 1 filter paper (Whatman, Kent, UK.). The filtrate was rotary evaporated to dryness at 25 °C (Bibby heated water bath, Heidolph Laborota 4000 motor unit condenser, Vacuubrand vacuum pump, Heidolph, Nurenberg, Germany). Dried extracts were stored under nitrogen at −20 °C before being analysed.

Water extracts were separated from their respective seaweed powders by centrifugation at 4500 rpm for 4 min due to sample viscosity. The supernatant was then frozen at −20 °C and freeze dried as before. Solid water extracts were stored under nitrogen at −20 °C.

A time study was carried out in water by extracting the seaweed powder according to the above method for 1, 2, 12, and 24 h. The 2 h crude water extract of the seaweed was also autoclaved at 121 °C for 15 min and tested for antimicrobial activity using the disk diffusion method.

### Antimicrobial activity of crude seaweed extracts against wound pathogens

The anti-MRSA activity of the extracts of the seaweeds *Fucus vesiculosus* and *Fucus serratus* were assessed using the CLSI standardised disk diffusion assay^42^ against clinically isolated pathogens donated by University Hospital Waterford. Among the clinical strains, there were two strains of MRSA (687, 688) containing the mecC gene that are reported to be present in Irish hospitals in very low levels. There was also a glycopeptide intermediate *S. aureus* (GISA) strain (689). The remainder of the strains were clinical mecA MRSA strains with varying antibiotic profiles. Dried extracts were aseptically dissolved in the solvent of their extraction at a concentration of 100 mg/mL. Disks were loaded with five 10 µL aliquots of this solution (5 mg/disc), allowing the disks to dry fully between loads. Negative control disks were loaded with 50 µL of the extraction solvent. 10 µg Chloramphenicol disks (Oxoid, Basingstoke, UK) were used as the positive control throughout this assay. A preliminary investigation comparing the dose to be used was undertaken by loading 1 mg, 3 mg and 5 mg on disks and comparing the data.

Strains were characterised by the National Methicillin - Resistant *Staphylococcus aureus* Reference Laboratory^43^. These strains were previously stocked in a 60:40 solution of sterile broth:glycerol and stored at − 20 °C. For antimicrobial testing, the bacterial strains were inoculated aseptically from their glycerine stocks at a concentration of 1:100 in Brain Heart Infusion broth (BHI, Oxoid Basingstoke, UK). The inoculated broth was allowed to incubate overnight at 37 °C.

After incubation, 1 mL of cultured broth was centrifuged at 13,000 rpm for 2 min to generate a cell pellet, the supernatant was discarded and the pellet re-suspended in 1 mL of sterile maximum recovery diluent (MRD). This was repeated a further two times to ensure that the cells were clean from metabolic waste before the adjustment of the bacteria to an optical density (OD_625_) of 0.10–0.12 (equivalent to 0.5 McFarland Standard or 10^7^–10^8^ colony forming units, cfu/mL).

A sterile swab was used to spread adjusted bacteria onto Mueller Hinton agar (MHA, Oxoid Basingstoke, UK) plates by swabbing the surface of the plates with culture within 15 min of adjustment, then rotating the plate 60 °C and spreading the bacteria before rotating a further 60 °C and spreading the bacteria again. The 5 mg disks prepared as per the disk diffusion method^44^, including the positive and negative controls, were transferred aseptically to the swabbed plates and allowed to chill in a refrigerator at 4 °C for 5 h to allow for diffusion of the extract on the disks into the agar. The plates were then incubated in the inverted position at the same temperature that the bacteria was originally grown as specified in Table 2.

### Minimum inhibitory concentration and minimum bactericidal concentration

The minimum inhibitory concentration (MIC) and minimum bactericidal concentration (MBC) of the extracts were tested using the CLSI standard broth dilution method^44^, with the modification that the plates would be read at 620 nm due to the limitations of the microtitre plate reader.

Dried extracts were dissolved to a starting concentration of 50 mg/mL in Mueller Hinton Broth (MHB). 100 µL of this solution was then added in triplicate wells of a 96 well microtitre plate, serial twofold dilutions were then carried out on these samples. A row of control samples were also prepared for each of the dilutions. Other control samples included three wells of 100 µL of 10 µg/mL chloramphenicol as a positive control, six wells of 100 µL MHB, three wells as a media only control and three wells as negative controls.

A 1% inoculation of MRSA (676) was prepared in BHI and allowed to incubate overnight at 37 °C. MRSA (676) was used as it was the most promising strain tested using the disk diffusion assay. The subsequent cells were then washed in triplicate with MRD and adjusted to a McFarland standard of 10^7^–10^8^ colony forming units per mL as described in section 2.2.4. The adjusted solution was then diluted 1:100 in MHB and 100 µL was loaded into each of the three rows of sample wells, the three positive control wells and the three negative control wells. 100 µL of sterile MHB was spiked into the remaining wells (sample controls, media only controls).

The plate was incubated overnight at 37 °C and subsequently read at 620 nm on a plate reader. This method was repeated in triplicate on different days. Minimum bactericidal concentration (MBC) was performed by spreading 50 µL from each well with the MIC reading onto MHA plates. The MBC is the lowest concentration which results in no growth after overnight incubation at 37 °C.

### Biofilm prevention cell viability analysis

Dried extracts were dissolved to a starting concentration of 50 mg/mL in Mueller Hinton Broth (MHB). 100 µL of this solution was then added in triplicate wells to a 96 well microtitre plate. Serial twofold dilutions were then carried out on these samples. A row of control samples were also prepared for each of the dilutions. Other control samples included three wells of 100 µL of 10 µg/mL chloramphenicol as a positive control, six wells of 100 µL of MHB, three as a media only control and three as a negative control.

A 1% inoculation of MRSA (676) was prepared in BHI and allowed to incubate overnight at 37 °C. The subsequent cells were then washed in triplicate with MRD and adjusted to a McFarland standard of 10^7^–10^8^ colony forming units per mL.The adjusted solution was then diluted 1:100 in MHB and was spiked 100 µL into each of the three rows of sample wells, the three positive control wells and the three negative control wells. 100 µL of sterile MHB was spiked into the remaining wells (sample controls, media only controls).

After overnight incubation at 37 °C, the supernatants were transferred to a separate plate. Each well of the original plate was washed in triplicate using sterile PBS and then spiked with 110 µL of MRD. The bottom of bacteria containing wells were carefully scraped into the MRD solution using 20–200 µL pipette tips. The resulting suspension was then carefully aspirated and transferred to a separate 96 well plate. Serial tenfold dilutions were made on bacteria containing samples and controls in MRD and then plated neat-10^−7^ to achieve plate counts. Positive and media only controls were plated neat only.

Plate counts were achieved for the supernatants using the same method. Biofilm prevention can then be calculated as a percentage against the negative controls. This method was repeated in triplicate on different days, agreement between results was taken that biofilms were scraped from the wells in a reproducible manner.

### Biofilm disruption cell viability analysis

A 1% inoculation of MRSA (676) was prepared as for the biofilm prevention cell viability assay. Subsequent to incubation for 48 h, treatments and controls were prepared for the wells as per the biofilm prevention cell viability assay. 100 µL of samples and controls then left to incubate for a further 18–20 h at 37 °C.

After overnight incubation at 37 °C, each well was washed in triplicate using sterile PBS and then spiked with 110 µL of MRD. Bacteria containing wells were carefully scraped into the MRD solution using 20–200 µL pipette tips. The resulting suspension was then carefully aspirated and transferred to a separate 96 well plate. Serial tenfold dilutions were made on bacteria containing samples and controls in MRD and then plated neat-10^−7^ to achieve plate counts. Positive and media only controls were plated neat only. Biofilm disruption can then be calculated as a percentage against the negative controls. This method was repeated in triplicate on different days.

### Crystal violet assay for biofilm disruption and prevention

The crystal violet assay performed was modified from Stepanovc *et al*.^45^. MRSA 676 was prepared and incubated as per the cell viability assays for both biofilm prevention and disruption,alternatively however, the cells were stained using crystal violet solution (Scichem, Cork, Ireland). The seaweed extract solution in the wells was then carefully removed and 200 µL of methanol (Sigma-Aldrich) was added to fix the biofilms. 100 µL of 1% crystal violet solution was added subsequent to removal of methanol and washing in PBS. The wells were then emptied and washed in triplicate using sterile water before the addition of 96% ethanol (Sigma-Aldrich) to solubilised the crystal violet. Finally, the plates were analysed using a microtitre plate reader at 570 nm.

## Conclusions

Extracts of *Fucus serratus* and *Fucus vesiculosus* were found to have a range of antimicrobial activity against clinically isolated MRSA including GISA and mecC strains. *Fucus vesisuclosus* water extracts also exhibited biofilm prevention and disruption activity, indicating a promising multitude of bioactivity which could potentially be utilized in therapeutics. Particularly, the combination of antibiofilm and antimicrobial activity was speculated to have potential value for use in a bioactive wound dressing setting.

## Data Availability

The authors confirm that the data supporting the findings of this study are available within this article.
